# Resolving the structure of V_3_O_7_·H_2_O and Mo-substituted V_3_O_7_·H_2_O

**DOI:** 10.1107/S2052520622006473

**Published:** 2022-07-13

**Authors:** Jürgen Schoiber, Daniela Söllinger, Volodymyr Baran, Thomas Diemant, Günther J. Redhammer, R. Jurgen Behm, Simone Pokrant

**Affiliations:** a Chemistry and Physics of Materials, University of Salzburg, Jakob-Haringer-Straße 2a, Salzburg 5020, Austria; b Maier-Leibnitz Zentrum MLZ Forschungsreaktor Munchen FRM-II, Lichtenbergstr. 1, Garching, Bavaria 85748, Germany; c Institute of Surface Chemistry and Catalysis, Ulm University, Albert-Einstein-Allee 47, 89081-Ulm, Germany; d Institute of Theoretical Chemistry, Ulm University, Albert-Einstein-Allee 11, 89081-Ulm, Germany; University of Antwerp, Belgium

**Keywords:** hydrated vanadate, vanadium oxide, powder neutron diffraction, powder X-ray diffraction, electrode material

## Abstract

Vanadate compounds, such as V_3_O_7_·H_2_O, are of high interest due to their versatile applications as electrode material for metal-ion batteries. In particular, V_3_O_7_·H_2_O can insert different ions such as Li^+^, Na^+^, K^+^, Mg^2+^ and Zn^2+^. In that case, well resolved crystal structure data, such as crystal unit-cell parameters and atom positions, are needed in order to determine the structural information of the inserted ions in the V_3_O_7_·H_2_O structure. In this work, fundamental crystallographic parameters, *i.e.* atomic displacement parameters, are determined for the atoms in the V_3_O_7_·H_2_O structure. Furthermore, vanadium ions were substituted by molybdenum in the V_3_O_7_·H_2_O structure [(V_2.85_Mo_0.15_)O_7_·H_2_O] and the crystallographic positions of the molybdenum ions and their oxidation state are elucidated.

## Abstract 2

This work provides detailed crystal structure data for V_3_O_7_·H_2_O. Furthermore, it gives insight into Mo-substituted V_3_O_7_·H_2_O.

## 1. Introduction

**Figure 1:**
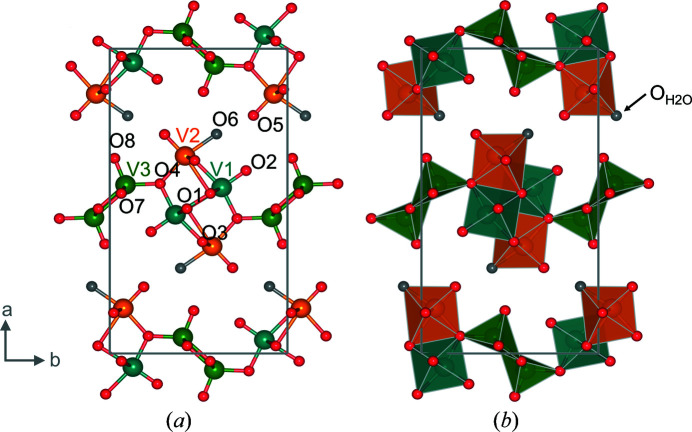
Crystal structure of V_3_O_7_·H_2_O based on data from Oka *et al.* (1990 ▸) viewed along the *c* axis, space group *Pnam*: (*a*) in ball-and-stick form and (*b*) polyhedron style. The colours turquoise (V1), orange (V2) and green (V3) represent the three different vanadium positions in the structure. Red spheres represent oxygen atoms. The grey spheres represent the oxygen of the water molecule in the structure.

Vanadate-based compounds are under investigation as potential next-generation electrode materials due to their ability to electrochemically insert various ions (Zhu *et al.*, 2014 ▸; Huang *et al.*, 2015 ▸; Moretti & Passerini, 2016 ▸; Yan *et al.*, 2016 ▸; Olszewski *et al.*, 2018 ▸). Therefore, interest arose from the battery materials community for the compound V_3_O_7_·H_2_O (also written as H_2_V_3_O_8_, HVO), which can intercalate Li^+^, Na^+^, K^+^, Mg^2+^ or Zn^2+^ (Simões *et al.*, 2014 ▸; Zhu *et al.*, 2014 ▸; Wang *et al.*, 2016 ▸; He *et al.*, 2017 ▸; Tang *et al.*, 2017 ▸; Rastgoo-Deylami *et al.*, 2019 ▸). This insertion behaviour is of high interest since there are only a few compounds that have this ability. Furthermore, the stability during electrochemical cycling is better for V_3_O_7_·H_2_O than for related materials, *e.g.* V_2_O_5_, due to hydrogen bonds in the structure (Gao *et al.*, 2009 ▸). Fig. 1 ▸ shows the crystallographic structure of V_3_O_7_·H_2_O determined by Oka *et al.* from powder X-ray diffraction (Oka *et al.*, 1990 ▸). The structure provides three symmetry-independent vanadium positions highlighted as coloured spheres [Fig. 1 ▸(*a*)], in turquoise (V1) with an oxidation state of +5, orange (V2) with an oxidation state of +4 and green (V3) with an oxidation state of +5, and with two different oxygen coordination chemistries [Fig. 1 ▸(*b*)].

So far, it has been suggested that one position (V3) has a square pyramidal coordination geometry, while two positions have octahedral ones (V1 and V2) (Oka *et al.*, 1990 ▸). However, the actual coordination geometries are still up for discussion. Considering bond valence sums, square pyramidal coordination rather than octahedral is present for the V2 position. This has also been stated by Rastgoo-Deylami *et al.* (Rastgoo-Deylami *et al.*, 2018 ▸). The possible position of the hydrogen atoms was assigned to an oxygen atom (O6) bonded to the V2 vanadium centre (Mettan *et al.*, 2015 ▸). This leads to the hydrogen-bonding scheme mentioned above, which interconnects the vanadium oxide layers. Very recently, a crystal data set for V_3_O_7_·H_2_O has been reported, including experimentally determined hydrogen-atom positions. However, it included fixed atomic displacement parameters for the various atom positions (Kuhn *et al.*, 2022 ▸). As shown in Fig. 1 ▸(*b*), an open sheet-like topology is present, where the insertion of different ions can occur between the V-site layers (Simões *et al.*, 2014 ▸; Zhu *et al.*, 2014 ▸; Wang *et al.*, 2016 ▸; He *et al.*, 2017 ▸; Tang *et al.*, 2017 ▸; Rastgoo-Deylami *et al.*, 2019 ▸). However, possible positions in the HVO structure have been determined experimentally or calculated (by DFT) so far for Li^+^, Mg^2+^ and Zn^2+^ (Kuhn *et al.*, 2022 ▸; Kundu *et al.*, 2018 ▸; Rastgoo-Deylami *et al.*, 2018 ▸). Li^+^ ions have a sixfold coordination, while Mg^2+^ ions seem to have square pyramidal coordination in the HVO structure, and tetrahedral and/or octahedral coordination was attributed to Zn^2+^ ions (Kuhn *et al.*, 2022 ▸; Rastgoo-Deylami *et al.*, 2018 ▸; Kundu *et al.*, 2018 ▸). These differences in coordination geometries hint at different insertion positions between the layers in the V_3_O_7_·H_2_O structure. Still, some questions remain unanswered. For example, as mentioned before, the position of the hydrogen atom in the structure is still under discussion. Furthermore, recently synthesized Mo-substituted HVO has been described, but it remains unclear whether Mo substitutes V sites randomly or whether specific unit-cell positions are favoured (Söllinger *et al.*, 2021 ▸). With this work, we are able to confirm the proposed position of the hydrogen atom in the V_3_O_7_·H_2_O structure from previous works and determine a complete crystallographic data set by correlating two diffraction data sets obtained by powder neutron and powder X-ray diffraction. In addition, the importance of atomic displacement parameters obtained for each atom on the different crystallographic positions is discussed in comparison to previously reported ones. Based on these results, we determined the Mo position in the V_3_O_7_·H_2_O structure for substituted materials and discussed the findings taking into account the oxidation state and the coordination chemistry of Mo.

## 2. Experimental

### 2.1. Hydro­thermal synthesis of V_3_O_7_·H_2_O and (V_1–*x*_Mo_*x*_)_3_O_7_·H_2_O

*Materials*. Vanadium(V) oxide (V_2_O_5_, 99.2%), anhydrous oxalic acid (H_2_C_2_O_4_, 98%) and l(+)-ascorbic acid (99+ %) were purchased from Alfa Aesar. Ammonium molybdate tetrahydrate [(NH_4_)_6_Mo_7_O_24_·4H_2_O, 99.98%] was purchased from Sigma Aldrich. All chemical reagents were used as received.

Compounds were synthesized according Söllinger *et al.* (2021 ▸).

*V_3_O_7_·H_2_O*. H_2_C_2_O_4_ (10.4 g) was dissolved in deionized water (100 ml) at room temperature. After complete dissolution of the oxalic acid, V_2_O_5_ (5 g) was added, followed by stirring for 3 h and further stirring at 80 °C for 5 h. The obtained solution was transferred into an evaporating dish and dried at 100 °C for 10 h followed by a calcination step at 400 °C for 10 h. The obtained as-synthesized V_2_O_5_ (VO) (2 g) was added to an aqueous ascorbic acid solution (20 ml, 0.025 *M*). The obtained suspension was stirred under reflux at 110 °C for 16 h in a round bottom flask. After this step, a hydro­thermal process was initiated. The whole suspension was transferred into an autoclave with a 100 ml polytetra­fluoro­ethyl­ene inlet. Additionally, deionized water (30 ml) was added to the suspension and brought to reaction at 220 °C for 6 h. The obtained precipitate was collected, washed with deionized water and iso­propanol, and dried at 80 °C for 3 h. Pristine V_3_O_7_·H_2_O without further modifications is denoted HVO in the following.

*(V_2.85_Mo_0.15_)O_7_·H_2_O*. Mo-substituted V_3_O_7_·H_2_O, was synthesized similar to HVO. To incorporate Mo^6+^ into VO, the soft chemistry process leading to VO was modified by adding an aqueous solution containing the Mo^6+^ ion. Therefore, (NH_4_)_6_Mo_7_O_24_·4H_2_O (1.77 g) was dissolved in deionized water (100 ml) to obtain an aqueous (0.1 *M*) Mo stock solution. To obtain 5 at% Mo in the V_3_O_7_·H_2_O structure, 4.75 g instead of 5 g V_2_O_5_ with Mo stock solution (27.5 ml, 0.1 *M*) were added to the oxalic acid solution and 9.88 g oxalic acid instead of 10.4 g were introduced to the precursor solution, respectively. The obtained compound with the formula (V_0.95_Mo_0.05_)_3_O_7_·H_2_O is labelled as HVO-Mo-5.

### 2.2. X-ray and neutron diffraction

#### 2.2.1. X-ray diffraction

The crystalline phases of the synthesized powders were studied by powder X-ray diffraction (PXRD) on a Bruker D8 Advance diffractometer with a goniometer radius of 280 mm with a fast-solid state LynxEye detector and an automatic sample changer. The measurements were carried out with Cu *K*α_1,2_ radiation in the 2θ range from 5° to 95° with a step size of 0.015°. All samples were prepared on zero-background single-crystal silicon sample holders. Full pattern refinement (Rietveld method) was performed with *FullProf* (Rodríguez-Carvajal, 1993 ▸). The three-dimensional visualization of the crystal structure was constructed via *VESTA* (Momma & Izumi, 2011 ▸).

#### 2.2.2. Neutron powder diffraction

The neutron diffraction (ND) experiment was performed at the Research Neutron Source Heinz Maier-Leibnitz (FRM II) Garching bei München, Germany, within the Rapid Access Program. Powder diffraction data were acquired in Debye–Scherrer geometry using the high-resolution powder diffractometer SPODI (Hoelzel *et al.*, 2012 ▸). Monochromatic neutrons with wavelength λ = 1.5482 Å were chosen from 551 reflection of a vertically focused composite germanium monochromator. The sample was contained in a 14 mm-diameter vanadium container and measured with constant rotation at 300 K. The high-resolution neutron powder diffraction data were collected in a 2θ range of 3° ≤ 2θ ≤ 152°, with a resolution step of 0.05°.

#### 2.2.3. Diffraction data evaluation

Data refinement was performed with the *FullProf* suite of programs. For V_3_O_7_·H_2_O: Final results were extracted from a joint refinement on neutron and X-ray diffraction data using equal weights. For (V_2.85_Mo_0.15_)O_7_·H_2_O: Mo-ion positions and the amount of occupancy were determined using XRD data only. The determined HVO structure from this work served as the starting structure model.

### 2.3. X-ray photoelectron spectroscopy analysis

The X-ray photoelectron spectroscopy (XPS) measurements were carried out at a PHI 5800 MultiTechnique ESCA System (Physical Electronics) using monochromated Al *K*α (1486.6 eV) radiation (250 W, 15 kV). The measurements were performed with a detection angle of 45° and a pass energy of 29.35 eV at the analyser to obtain detailed spectra. The samples were neutralized with electrons from a flood gun (current 3 µA) to compensate for charging effects at the surface. For binding energy calibration, the C(1*s*) peak was set to 284.8 eV. The peak fit of the detail spectrum in the Mo(3*d*) region was done with *CasaXPS* (Walton *et al.*, 2010 ▸), using a Shirley-type background and a peak doublet (Gaussian–Lorentzian peak shape, intensity ratio 3:2, spin-orbit splitting 3.1 eV).

## 3. Results and discussion

### 3.1. The V_3_O_7_·H_2_O structure

V_3_O_7_·H_2_O was first described by Oka *et al.* (1990 ▸). Rastgoo-Deylami *et al.* (2018 ▸) published a resolved HVO structure, again, but still without any hydrogen positions, which had been proposed earlier by Mettan *et al.* (2015 ▸). Although Mettan *et al.* (2015 ▸) performed structure refinements on neutron diffraction data acquired at 4 K and 300 K. Moreover, no crystallographic data set was published in that work. Recently, Kuhn *et al.* (2022 ▸) evaluated lithium-inserted HVO and determined only one hydrogen position, bonded to the same oxygen stated by Mettan *et al.* (2015 ▸). Hence, our article aims to re-evaluate the work by combining ND and XRD powder patterns and providing a crystallographic data file (see supporting information).

**Figure 2:**
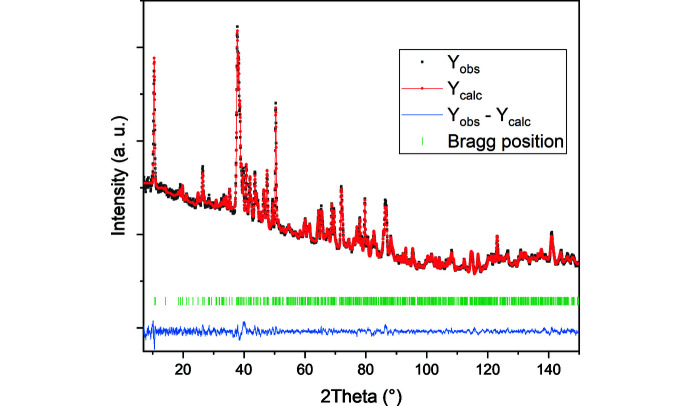
Neutron diffraction (ND) pattern of Rietveld refined V_3_O_7_·H_2_O. The black dots represent the experimentally obtained ND pattern. The red line with dots represents the calculated pattern. The blue line shows the difference between the experimental and calculated patterns. The green vertical lines represent the Bragg peak positions.

**Figure 3:**
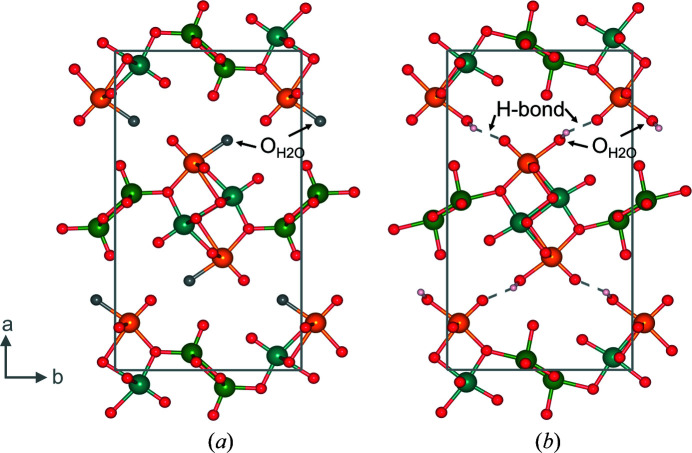
Crystal structure of V_3_O_7_·H_2_O with view along the *c* axis, space group *Pnam*, in ball and sticks form without (*a*) and with hydrogen position (*b*); data sets for (*a*) are from Oka *et al.* (1990 ▸); data sets for (*b*) are from the refined structure. The colours turquoise (V1), orange (V2) and green (V3) represent the three different vanadium positions in the structure. Red spheres represent oxygen atoms, while the grey sphere represents the oxygen atom of the crystal water molecule without hydrogen bonds. The pinkish spheres represent the hydrogen atoms of the water molecule in the structure.

Fig. 2 ▸ shows the obtained pattern from ND Rietveld refinement yielding an *R*_Bragg_ value of 4.6% in space group setting *Pnam*, which was originally proposed by Oka *et al.* (1990 ▸). The refinement led to the structure presented in Fig. 3 ▸.

Fig. 3(*a*) shows the HVO structure determined by Oka *et al.* (1990 ▸) without the hydrogen-atom position determined. In this case, we highlighted the oxygen of the crystal water molecule in grey to symbolise the missing H bonds. In comparison, the structure we obtained from Rietveld analysis from combined ND and XRD results is shown in Fig. 3(*b*). Note that we kept the stated coordination chemistry from Oka *et al.* (1990 ▸) with V1 (turquoise) and V2 (orange) in octahedral and V3 (green) in square pyramidal coordination.

The basic unit of the structure is a band of V1 and V2 octahedra. The V1 octahedra are connected to each other by sharing two edges forming a non-planar zigzag chain extending in **c** direction. The V2 octahedra share three edges with V1 octahedra and are laterally attached to this chain along the **a** direction. The V3 sites are described to have a square pyramidal oxygen coordination; by sharing two edges within the base-plane of the pyramid, they form a zigzag chain with the apex of the pyramids alternately pointing up and down, and which interconnects the V1–V2 band to a layer of V sites extending in the **b** **c** plane. The hydrogen atoms are bonded to the O6 oxygen atom of the V2 octahedron and interconnect these layers via hydrogen bonding to the O5 oxygen atom of a V2 octahedron of the next layer, stacked along the crystallographic *a* axis.

The positions of the three vanadium atoms in the present refinement of the HVO structure show less distorted octahedra than in the result from Oka *et al.* (1990 ▸), leading to a smaller variance of the different bond lengths (Table 1 ▸). In particular, the square pyramidal coordination chemistry of the V3 position, hence, the bond lengths between V3 and surrounding oxygen atoms, changes substantially compared to the initially proposed structure of Oka *et al.* (1990 ▸). The bond length between V3 and O7 changes from 1.8975 (3) to 1.82644 (5) Å, which is the bond in the square plane of the pyramidal geometry, while the axial bond between V3 and O8 changes from 1.5824 (13) to 1.86009 (6) Å. Comparing the positions of V1 and V2, bond lengths between O1 and V1/V2 and O2 and V1 (Table 1 ▸) show substantial changes, too. Additionally, the positions of the vanadium atoms V1 and V2 are less displaced from the centre of the coordination octahedron. Overall, the obtained bond lengths between the vanadium centres and the surrounding oxygens are typical for (hydrated) vanadates (Liu *et al.*, 2010 ▸; Wu *et al.*, 2009 ▸). The distance between V2 and O1 is in general the longest compared to all other bonds in te structure. It changed from 2.4731 (9) Å [structure from Oka *et al.* (1990 ▸)] to 2.20580 (5) Å (this work). This distance is at the limit of attributing the two atoms to a bond or not. As seen in Fig. 3 ▸, we decided to create awareness of the potential chemical interaction between these sites and present it as a bond and the coordination of the V2 atom as a distorted octahedron.

Evaluating the position of the hydrogen atoms in the structure by residual electron/nuclear density analysis, we determined from experimental data the same crystallographic position for the hydrogen atom at the oxygen atom O6 as Mettan *et al.* (2015 ▸). However, Mettan *et al.* (2015 ▸) discussed a second position of the H atom attached to the same O6 atom, since a value of 0.6 was found for hydrogen occupancy, indicating that slightly more than a half of the position was occupied by hydrogen atoms. We obtained similar results. In the work of Mettan *et al.* (2015 ▸), no values for atomic displacement parameters (*U*) were mentioned but set to be equal for all atoms. In this work, we determined isotropic displacement parameters (*U*_iso_) by combining ND and XRD patterns during the Rietveld refinement for all independent atomic positions present. With that, we obtained varying *U*_iso_ values, listed in Table 2 ▸, for the vanadium, oxygen and hydrogen positions. By doing so, the occupancy value of the hydrogen atom changed to 1 after evaluating reasonable *U*_iso_ values (see Table 2 ▸). In particular, oxygen atoms with multifold bond situations (three to four) have lower *U*_iso_ values than those with fewer bonds. These values are in good agreement with the condition that strongly bonded oxygen atoms, *e.g.* O1, O3 or O4, are locked into their particular crystallographic positions. In contrast, less connected oxygens, *i.e.* O2, O5 and O8, will vibrate more strongly around the given position. These results are in contrast to the previous results of Kuhn *et al.* (2022 ▸), who kept *U*_iso_ values constant for each atom species. Hence, the obtained values for isotropic displacement parameters give new insight into the oxygen positions in the HVO structure and show the dependence on the multifold bond situation.

Interestingly, the H atom shows a very high *U*_iso_ value. Since Mettan *et al.* described that there are two possible positions for the hydrogen atom, as mentioned above, a high *U*_iso_ value might also indicate that the hydrogen atoms of the water molecule cannot be precisely located and shows some positional disorder, possibly induced by the hydrogen-bond configuration. Hence, while a full occupancy of the hydrogen atom at the determined position was obtained, an exact position cannot be stated because of the high isotropic atom displacement parameter.

### 3.2. The Mo-substituted V_3_O_7_·H_2_O structure

In order to carry out Rietveld analysis, an excellent starting crystal structure data set is necessary if structurally analogous, chemically modified compounds, or crystal structure changes during (electro)chemical processes are investigated. We demonstrate the necessity and potential of using the obtained HVO crystallographic data set in that context.

**Figure 4:**
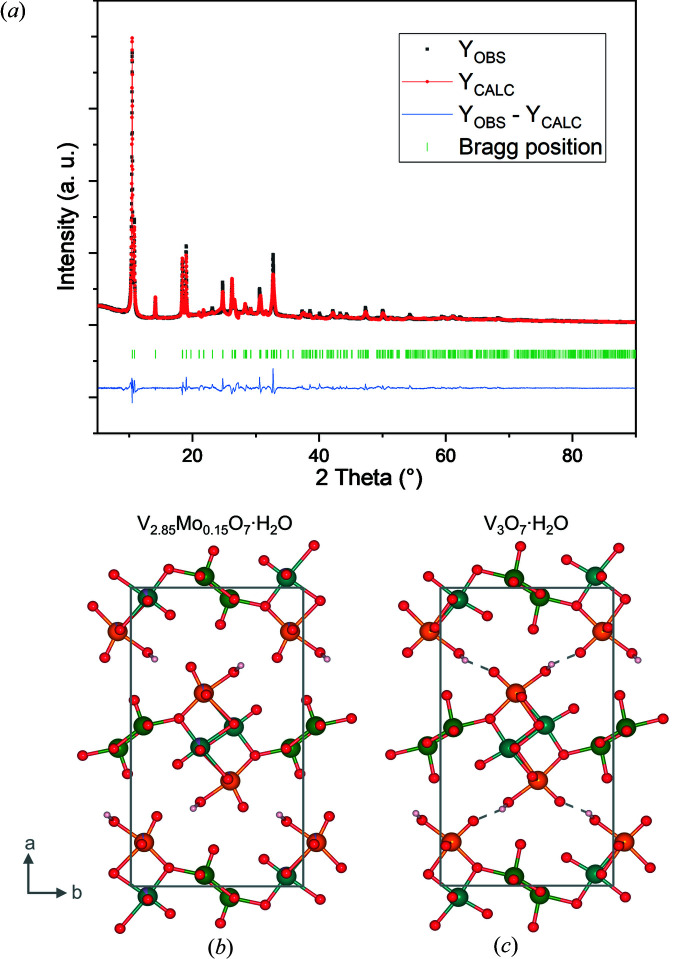
(*a*) XRD powder pattern of (V_2.85_Mo_0.15_)O_7_·H_2_O. The black dots represent the obtained XRD pattern. The red line with dots represents the calculated pattern. The blue line shows the difference between the experimental and calculated patterns. The green vertical lines represent the Bragg peak positions. (*b*) Refined Mo-substituted HVO structure (V_2.85_Mo_0.15_)O_7_·H_2_O. (*c*) HVO structure.

Fig. 4 ▸(*a*) shows the obtained refinement plot of (V_2.85_Mo_0.15_)O_7_·H_2_O using starting values from our HVO structure file. We obtained a fit with an *R*_Bragg_ of 7.07% and a reliable structure [Fig. 4 ▸(*b*)]. Unit-cell parameters of refined HVO and Mo-substituted HVO, as well as from literature, are summarized in Table 3 ▸. The obtained parameters for HVO are between the previously reported ones; *e.g.* we obtained a value of 16.865 (5) Å for unit-cell parameter *a*, while Oka *et al.* (1990 ▸) and Rastgoo-Deylami *et al.* (2018 ▸) reported 16.930 and 16.844 Å, respectively. Comparing the unit-cell parameters of Mo-substituted HVO with unsubstituted HVO, an increase of the unit-cell parameters is observed. This increase corroborates the results of a recent study (Söllinger *et al.*, 2021 ▸).

Our attempts to refine the Mo-substituted structure starting with values obtained from Oka *et al.* (1990 ▸) or Rastgoo-Deylami *et al.* (2018 ▸) failed as soon as atomic positions were refined. Specifically, the position O2 shifted towards the V1 position, resulting in a V1–O2 distance below 1 Å, which is unreasonable. This shift is observed in the refinement with the novel HVO data set, too. Still, a reasonable bond distance was obtained compared to refinements with starting data sets from Oka *et al.* (1990 ▸) or Rastgoo-Deylami *et al.* (2018 ▸) (see Table 1 ▸). Hence, the interaction between these two positions became more robust with the substitution of Mo, leading to a shorter bond distance.

Additionally, with the previously published HVO structures from Oka *et al.* or Rastgoo-Deylami *et al.* (2018 ▸), no reliable model for the Mo occupation at any of the three possible sites could be determined. However, with our set of parameters, Mo ions were detected at the V1 and V2 sites. An all-over Mo-substitution of 5.2 at% was derived, resulting in the chemical composition of (V_2.842_Mo_0.158_)O_7_·H_2_O. This Mo atomic concentration is close to the target value of the synthesis strategy, that is 5 at% substitution of vanadium with molybdenum. Furthermore, the result indicates that the combined refinement of X-ray and neutron diffraction data yields reliable results. Around two-thirds of the Mo content are present at the V1 site, while the other third is located at the V2 site. No Mo-content could be detected at the V3 site. Furthermore, the hydrogen bond broke as the oxygen atom from the O5 position shifted away from the coordinated water molecule’s hydrogen atoms.

**Figure 5:**
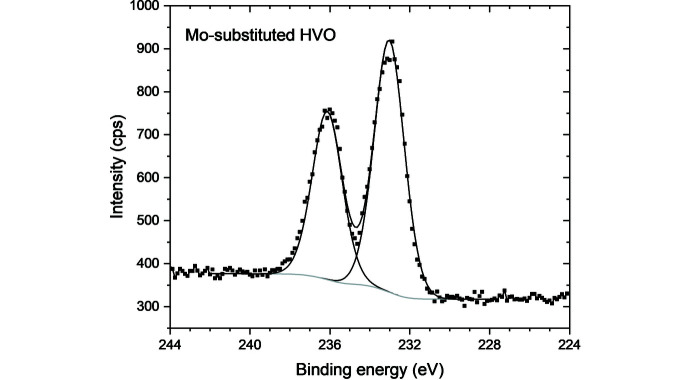
XPS spectrum of Mo-substituted HVO in the Mo(3*d*) domain.

Since mild reductive synthesis procedures were applied, we also checked for the possibility that, in addition to the reduction of V^5+^ to V^4+^ species, Mo^6+^ was reduced to Mo^5+^ or lower. Hence, the oxidation state of Mo in the HVO structure was determined by XPS analysis. The detail of the spectrum in the Mo(3*d*) region (Fig. 5 ▸) shows a single peak doublet at 233.0/236.1 eV, which we assign to Mo^6+^ (Moulder *et al.*, 1992 ▸).

Hence, no reduction of Mo^6+^ occurs during the synthesis. Mo^6+^ occupies preferentially the octahedrally coordinated V1 (V^5+^) and V2 (V^4+^) sites (Shannon, 1976 ▸) instead of the pyramidally coordinated V3 (V^5+^) site. This preference for octahedral coordination cannot be explained based on simple steric arguments alone, since the ionic radius difference between V^5+^ and Mo^6+^ is similar for octahedral and pyramidal coordination (Table 4 ▸). We believe that quantum chemical calculations are necessary to elucidate this preference.

## 4. Conclusion

Phase pure V_3_O_7_·H_2_O and (V_2.85_Mo_0.15_)O_7_·H_2_O compounds were synthesized via a hydro­thermal synthesis route. The pristine vanadate compound was investigated by powder ND and XRD techniques. We resolved the hydrogen position in the crystal structure, leading to an parameter set which has the potential to be used for further Rietveld analysis of modified compounds. We demonstrated this by investigating (V_2.85_Mo_0.15_)O_7_·H_2_O by XRD. Our data set led to reasonable structural refinements. This allowed us to determine the Mo content in the structure and evaluate the preferred site occupancy of the substituting ion. Furthermore, we could evaluate the oxidation state of molybdenum, namely +6. As an outcome of this work, we provide a high-quality structure file that can be used to analyse further V_3_O_7_·H_2_O-related mechanisms or compounds, such as ion-insertion mechanisms or vanadium substitution.

## Supplementary Material

Crystal structure: contains datablock(s) global, VO, VMoO. DOI: 10.1107/S2052520622006473/je5048sup1.cif

Rietveld powder data: contains datablock(s) VO. DOI: 10.1107/S2052520622006473/je5048VOsup2.rtv

Rietveld powder data: contains datablock(s) VMoO. DOI: 10.1107/S2052520622006473/je5048VMoOsup3.rtv

CCDC references: 2181268, 2181269

## Figures and Tables

**Table 1:** Bond lengths (in Å) between vanadium and oxygen atoms

		V_3_O_7_·H_2_O			(V_2.85_Mo_0.15_)O_7_·H_2_O
V*x*	O*y*	Oka *et al.* (1990 ▸)†	Rastgoo-Deylami *et al.* (2018 ▸)†	This work	This work
V1	O1	1.9470 (3)	1.9425 (16)	1.84584 (5)	1.82951 (10)
	O2	1.5900 (10)	1.581 (5)	1.81928 (5)	1.55687 (10)
	O3 ax‡	2.0718 (8)	2.067 (5)	2.02833 (5)	2.350014 (12)
	O4 ax	1.9101 (11)	1.897 (5)	1.95870 (5)	1.98430 (11)
V2	O1 ax	2.4731 (9)	2.460 (5)	2.20580 (5)	2.38819 (11)
	O3	1.9068 (3)	1.8904 (13)	1.85642 (5)	1.82911 (10)
	O4	2.0288 (11)	2.019 (5)	2.01154 (5)	1.96378 (9)
	O5 ax	1.6058 (10)	1.597 (5)	1.67657 (4)	1.62805 (10)
	O6	2.0557 (9)	2.044 (5)	2.01568 (5)	2.00464 (9)
V3	O4	1.8159 (9)	1.806 (4)	2.05096 (7)	1.97249 (11)
	O7	1.8975 (3)	1.8793 (14)	1.82644 (5)	1.83821 (10)
	O8 ax	1.5824 (13)	1.573 (5)	1.86009 (6)	1.53280 (10)

† Results obtained from available cif-file from the ICSD.

‡ ax means the oxygen atom O*y* is in axial coordination to the vanadium central atom.

**Table 2:** Isotropic displacement parameters of hydrogen, vanadium and oxygen atoms in the V_3_O_7_·H_2_O structure

Atom	No. of bonds	*U*_iso_ value (Å^2^)
H1	1	0.09780
V1	6	0.02398
V2	6	0.01384
V3	5	0.01281
O1	4	0.00820
O2	1	0.01289
O3	3	0.01182
O4	3	0.00924
O5	1	0.01412
O6	3	0.01238
O7	3	0.0065
O8	1	0.01731

**Table 3:** Unit-cell parameters of (Mo-substituted) V_3_O_7_·H_2_O structures

	V_3_O_7_·H_2_O			(V_2.85_Mo_0.15_)O_7_·H_2_O
Unit-cell parameter	Oka *et al.* (1990 ▸)†	Rastgoo-Deylami *et al.* (2018 ▸)† ‡	This work	This work
*a* (Å)	16.92979 (20)	16.844 (1)	16.8650 (5)	16.8790 (12)
*b* (Å)	9.3589 (1)	9.309 (1)	9.3290 (3)	9.3325 (5)
*c* (Å)	3.64432 (4)	3.625 (1)	3.6332 (8)	3.64357 (19)
*V* (Å^3^)	577.42	568.40	571.96 (3)	573.95 (6)

† Results obtained from available cif-file from the ICSD.

‡ The values have been transposed from *Pnma* to *Pnam* for better comparison.

**Table 4:** Ionic radii of selected vanadium and molybdenum ions

Species	Radii in CN† 5 (Å)	Radii in CN 6 (Å)
V^4+^	n.r.‡	0.58
V^5+^	0.46	0.54
Mo^6+^	0.5	0.59

† CN means coordination number.

‡ n.r. means not relevant due to the coordination situation of the ion in the HVO structure.
